# A combinatorial method to visualize the neuronal network in the mouse spinal cord: combination of a modified Golgi-Cox method and synchrotron radiation micro-computed tomography

**DOI:** 10.1007/s00418-020-01949-8

**Published:** 2021-01-04

**Authors:** Liyuan Jiang, Yong Cao, Xianzhen Yin, Shuangfei Ni, Miao Li, Chengjun Li, Zixiang Luo, Hongbin Lu, Jianzhong Hu

**Affiliations:** 1 Department of Spine Surgery, Xiangya Hospital, Central South University, Xiangya Road No. 87, Changsha, 410008 Hunan People’s Republic of China; 2 Key Laboratory of Organ Injury, Aging and Regenerative Medicine of Hunan Province, Changsha, 410008 People’s Republic of China; 3 Department of Sports Medicine, Xiangya Hospital, Central South University, Xiangya Road No. 87, Changsha, 410008 Hunan People’s Republic of China; 4 Xiangya Hospital-International Chinese Musculoskeletal Research Society Sports Medicine Research Centre, Changsha, People’s Republic of China; 5 Hunan Engineering Research Center of Sport and Health, Changsha, People’s Republic of China; 6 Center for Drug Delivery System, Shanghai Institute of Materia Medica, Chinese Academy of Sciences, Shanghai, 201203 People’s Republic of China

**Keywords:** SRμCT, Modified Golgi-Cox method, Spinal cord, Neuronal network, Three-dimension

## Abstract

Exploring the three-dimensional (3D) morphology of neurons is essential to understanding spinal cord function and associated diseases comprehensively. However, 3D imaging of the neuronal network in the broad region of the spinal cord at cellular resolution remains a challenge in the field of neuroscience. In this study, to obtain high-resolution 3D imaging of a detailed neuronal network in the mass of the spinal cord, the combination of synchrotron radiation micro-computed tomography (SRμCT) and the Golgi-cox staining were used. We optimized the Golgi-Cox method (GCM) and developed a modified GCM (M-GCM), which improved background staining, reduced the number of artefacts, and diminished the impact of incomplete vasculature compared to the current GCM. Moreover, we achieved high-resolution 3D imaging of the detailed neuronal network in the spinal cord through the combination of SRμCT and M-GCM. Our results showed that the M-GCM increased the contrast between the neuronal structure and its surrounding extracellular matrix. Compared to the GCM, the M-GCM also diminished the impact of the artefacts and incomplete vasculature on the 3D image. Additionally, the 3D neuronal architecture was successfully quantified using a combination of SRμCT and M-GCM. The SRμCT was shown to be a valuable non-destructive tool for 3D visualization of the neuronal network in the broad 3D region of the spinal cord. Such a combinatorial method will, therefore, transform the presentation of Golgi staining from 2 to 3D, providing significant improvements in the 3D rendering of the neuronal network.

**Supplementary Information:**

The online version contains supplementary material available at 10.1007/s00418-020-01949-8.

## Introduction

The spinal cord, which transmits descending and ascending neural signals, is an essential component of the central nervous system. Neurons, as the main functional units of the spinal cord, are responsible for receiving and transmitting signals (Lovinger 2008). Hence, exploring the morphology of neuronal networks is important to understand the normal function of the spinal cord and the pathogenesis of related disorders (Hogstrom et al. 2016; Xu et al. 1993).

Histological staining techniques are widely used in research on neuronal morphology (Tsai et al. 2003). However, these methods mainly rely on two-dimensional (2D) histological sections. Unfortunately, 2D images of neurons can lead to data misinterpretation because they do not capture morphological information in the third dimension (Parekh and Ascoli 2013; Cedola et al. 2017). Although researchers achieved limited 3D imaging of neural structure using confocal microscopy, it is still challenging to visualize the microstructure of large-size samples using confocal microscopy (St Croix et al. 2005). Therefore, neuroscience research needs an imaging tool that can help to reveal the 3D neuronal networks of a broad range of samples.

Synchrotron radiation micro-computed tomography (SRμCT) has high resolution and is particularly suitable for 3D imaging of the microstructure of low-absorbing biomedical samples (Strotton et al. 2018; Saccomano et al. 2018; Tesarova et al. 2019). This technique has been used for 3D neuroimaging of the spinal cord. In our previous studies, SRμCT was proposed for 3D imaging of intramedullary microvessels (Cao et al. 2015, 2017; Hu et al. 2014, 2015; Wu et al. 2018). In addition, Fratini et al. reported that they achieved 3D imaging of the microvascular network and the neurons in the unstained mouse spinal cord using SRμCT (Fratini et al. 2015). Later, Bukreeva. et al. performed a quantitative investigation of the 3D neuronal network in the mouse spinal cord (Bukreeva et al. 2017). However, in these studies, the visualized 3D neurons were not intact, and morphological information of axons and dendrites was almost completely lacking. This is because axons and dendrites have a very similar refractive index to the extracellular matrix, producing insufficient contrast to define the outlines of the neurites. Therefore, it is challenging to distinguish axons and dendrites from the extracellular matrix in the unstained spinal cord tissue. 3D imaging of a single neuron in its entirety is a very desirable research goal. Increasing the contrast of the neuronal features is the key to visualizing the entire 3D architecture of neurons through SRμCT.

The Golgi staining technique, based on the impregnation of neural tissue with a heavy metal precipitate, is a classic neuronal staining technique that provides a high-resolution view, allowing all morphological features of the neuron to be visualized (Kassem et al. 2018; Parekh and Ascoli 2013). The heavy metal precipitate is deposited in the somas, axons, and dendrites of neurons, increasing their absorption contrast from the extracellular matrix, which helps outline individual neurons in their entirety. A previous study reported 3D imaging of the human cortex using a combination of conventional micro-CT and Golgi labeling(Mizutani et al. 2008). However, excessive sample artefacts and incomplete vasculature largely prevented the full 3D structure of single neurons from being clearly identified. Hence, a modified Golgi staining method is necessary for improved 3D visualization of neurons. A commercially available kit that is based on Golgi-Cox impregnation and is widely used in laboratories. However, when the procedure was performed according to the current standard manufacture’s protocol, staining usually results in different degrees of background staining, numerous artefacts, and incomplete vasculature. In the present study, we optimized the Golgi-Cox method (GCM) and developed a modified GCM (M-GCM) that can further clear the background, reduce the density of artefacts, and incomplete vascular structure. Moreover, we further combined the M-GCM and SRμCT to achieve improved 3D imaging of neuronal networks in a mass of the mouse spinal cord. The combinatorial method shown here does neither require a destructive sample preparation procedure, then allowing the 3D imaging of the neuronal network in a mass of mouse spinal cord matrix. It will further promote the progress of the visualization of the neuromorphology.

## Material and methods

### Experimental animals and ethics statement

All research protocols were approved by the Animal Ethics Committee of Central South University. Animal care and use were conducted under the guidelines of the Administration Committee of Affairs Concerning Experimental Animals in Hunan Province, China. A total of 24 adult male C57/BL6 mice weighing approximately 20–23 g each were obtained from the Animal Center of Central South University and were kept in a temperature-controlled room with a 12/12-h light/dark cycle with food and water ad libitum. All mice were randomly divided into two groups, the GCM group (*n* = 12) and the M-GCM group (*n* = 12). In each group, eight mice were used for Golgi staining and SRμCT detection and histology (*n* = 8). Four mice were used for residual blood comparison (*n* = 4).

### Comparison of fresh spinal cord tissue

To confirm M-GCM could completely remove the blood in vessels, the fresh spinal cord tissue was used for comparison between two groups before the Golgi impregnation. In the GCM group, four experimental mice were deeply anesthetized with ketamine (100 mg/kg, intraperitoneal injection)/ xylazine (10 mg/kg, intraperitoneal injection) and were euthanized using a CO2 chamber (CO2 flow rate: 3 L/min). Continue CO_2_ until 1 min after breathing stops. After euthanasia, the thoracic spinal cord from four randomly selected mice was directly removed and rinsed in the double-distilled water (dd-H_2_O). In the M-GCM group, another four randomly selected mice were perfused with 100 ml artificial cerebrospinal fluid after euthanasia (ACSF: dd-H_2_O 1000 ml, NaCl 125 mM, KCl 3 mM, CaCl2 2.5 mM, MgSO_4_ 1.3 mM, NaH_2_PO_4_ 1.25 mM, NaHCO_3_ 26 mM, Glucose 13 mM) until blood was utterly flushed out, which took up to 5 min, and then the spinal cord was removed. After that, the fresh spinal cord tissue in the two groups were both sectioned sagittally using a freezing microtome (Leica). The thickness of the sections was 200 μm. Sections were observed using the stereomicroscope and 40 × were captured for comparison.

## Golgi-Cox impregnation

### The GCM group

An FD Rapid GolgiStain Kit (FD NeuroTechnologies, INC., Catalog: PK401) was used in the present study. Golgi staining was performed according to the current standard protocol (Du 2019). Briefly, the impregnation solution (Solution A/B) was prepared by mixing equal volumes of Solutions A and B at least 24 h before use. Eight experimental mice were euthanized after deeply anesthetized. After euthanasia, we carefully removed the thoracic spinal cord and quickly rinsed the tissue in dd-H_2_O to remove the blood from the surface. The spinal cord was equally divided into two segments (length: 5 mm; diameter: 1.5 mm). One was used for SRμCT detection and the other for histology. The tissue was immersed in the impregnation solution (Solution A/B, 1:1), and stored at room temperature for 2 weeks in the dark. The impregnation solution was replaced after the first 12 h of immersion or the next day; the tissue was then transferred into Solution C and stored at room temperature in the dark for 72 h. Solution C was replaced at least once after the first 24 h of immersion or the next day. After the above procedures were performed, the spinal cord tissue was dehydrated using a graded ethanol series (70, 80, 90, 95, 100%) at room temperature. Finally, the spinal cord tissue was maintained in 100% ethanol until SRμCT detection. The detailed procedures are shown in the flow chart (Fig. S1).

### The M-GCM group

An FD Rapid GolgiStain Kit was used in this group. Eight mice were euthanized using a CO2 chamber as above mentioned. Subsequently, the mice were perfused with 100 ml ACSF. Then the thoracic spinal cord tissue was quickly removed and equally divided into two segments. One was used for SRμCT detection and the other for histology. The tissue was immersed in the impregnation solution (Solution A/B, 1:1) and stored at room temperature for 2 weeks in the dark. The impregnation solution was replaced after the first 12 h. Then, the spinal cord tissue was rinsed in dd-H_2_O for 24 h. The dd-H_2_O was replaced after the first 12 h. The spinal cord tissue was transferred into Solution C and stored at room temperature in the dark for 72 h. Solution C was replaced at least once after the first 24 h. Next, the spinal cord tissue was rinsed in dd-H_2_O for another 24 h. After all the above procedures were completed, the spinal cord tissue was dehydrated using a graded ethanol series (70, 80, 90, 95, 100%) at room temperature. Finally, the spinal cord tissue was maintained in methyl salicylate (Millipore Sigma, M6752-1L) for at least 72 h before SRμCT detection.

### Tissue processing and microscopic examination

The Golgi-Cox staining spinal cord tissue was sectioned sagittally or coronally (120 μm) using a freezing microtome (Leica).

*The GCM group *Three sections from each mouse were selected for histological analysis. The selected sections were mounted on a gelatin-coated microscope slide. Solution D/E was prepared; this solution consisted of one part Solution D, one part Solution E, and two parts dd-H_2_O. The sections were rinsed twice in dd-H_2_O two times, placed in the staining solution (Solution D/E) for 5 min, and then rinsed in dd-H_2_O two more times. The sections were dehydrated in sequential rinses of 50, 75, 95, and 100% ethanol for 4 min each. The sections were then cleared in xylene three times for 5 min each. The sections were coverslipped with Eukitt®quick-hardening mounting medium. An Olympus BX51 microscope equipped with Amscope MU1003 18MP CMOS USB 3.0 digital color camera (Olympus, Tokyo, Japan) was used for microscopic examination. The 40 ×, 100 ×, 200 ×, and 600 × images were captured for analysis. The exposure time was 20–80 ms.

*The M-GCM group *The selected sections were rinsed in dd-H_2_O for 5 min, placed in the staining solution (Solution D/E) for 5 min, and then rinsed in dd-H_2_O for 5 min. The sections were dehydrated in sequential rinses of 50, 75, 95, and 100% ethanol for 4 min each rinse. Next, the sections were mounted on a gelatin-coated microscope slide and left to air-dry for 2 min at room temperature. The sections were cleared in methyl salicylate for half an hour and then coverslipped. The microscopic examination was performed in the same way as for the GCM group.

### Comparison of the photomicrograph

Three photomicrographs of sagittal sections were selected from each mouse for comparison (*n* = 3 × 8).

*Stained vasculature comparison:* To confirm fewer blood vessels were stained in the M-GCM group, 10 × and 20 × images of sagittal sections (including the area of white matter and gray matter) were captured and compared between the two groups.

*Background comparison:* To confirm that the M-GCM reduced the background staining, an area with the same size (30 μm × 45 μm) located at the unstained background from the 60 × images in each sample were randomly selected for the comparison of background staining. And the gray value of the background was identified using image J ver. 1.6 (NIH, Bethesda, MD, USA).

*Artefact comparison:* To confirm the M-GCM generated fewer artefacts (object does not have discernible dendrites or axons), we captured continuous 20 × images (*n* = 40) located at the gray matter from different sections. The step size was 2 μm. Then the 3D reconstruction was performed using Imaris 9.2 ver. (Imaris Bitplane, Switzerland). Then, artefacts in the 3D volume (740 μm × 900 μm × 80 μm) were manually counted.

### X-ray radiation damage analysis

To confirm whether there was X-ray radiation damage on the cellular morphology, we cut the scanned spinal cord into sections. We captured continuous 20 × images and located at the gray matter and performed 3D reconstruction using Imaris 9.2 ver as above mentioned. Then, we performed a comparison of neuronal morphology between scanned tissue and unscanned tissue.

### High-resolution SRμCT detection

**Fig. 1:**
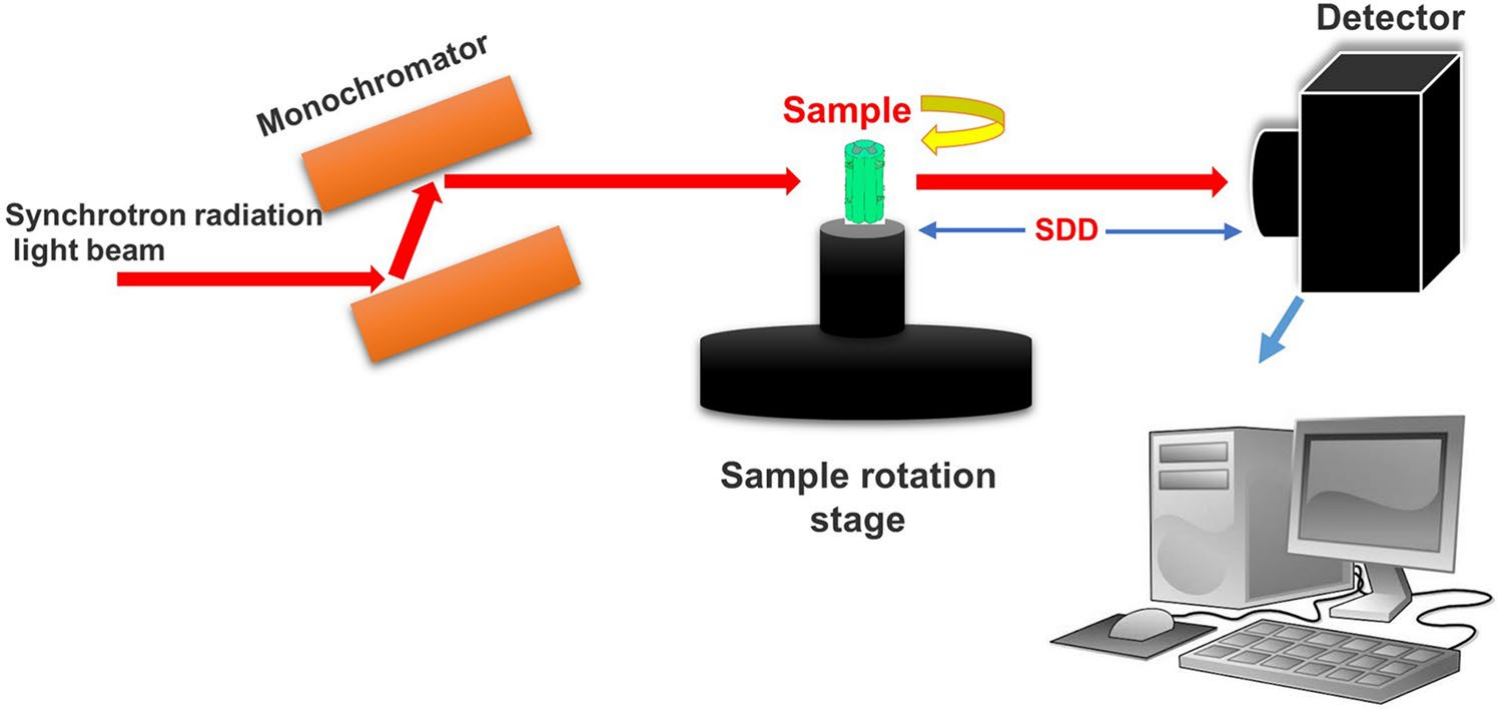
Schematic depiction of beamline experimental station at the BL13W1 at the Shanghai Synchrotron Radiation Facility (SSRF) in China. The samples were fixed on a sample rotation stage. The images were collected by a detector located at a 3.5 cm distance from the sample after transmission of a monochromatic synchrotron radiation X-ray beam through the sample and delivery to the image acquisition system

Specimens were scanned at the BL13W1 beamline of the Shanghai Synchrotron Radiation Facility (SSRF, China). A schematic depiction of beamline experimental station is shown in Fig. 1. The samples were fixed in the middle of the sample stage. The samples were scanned with a photon energy of 20.0 keV. The size of the beam was approximately 45 mm (horizontal) × 5 mm (vertical), and a double-crystal monochromator, with Si (111) and Si (311) crystals, was used to monochromatize the X-rays. After penetration through the sample, X-rays were converted into visible light with a cleaved Lu_2_SiO_5_: Ce single-crystal scintillator (10 μm thickness). Projections were magnified by diffraction-limited microscope optics with × 10 magnification and digitized with a high-resolution detector (ORCA Flash 4.0 Scientific CMOS, Hamamatsu K.K., Shizuoka Prefecture, Japan) with a physical pixel size of 0.65 μm × 0.65 μm. The samples were rotated continuously during scanning, and 900 projection images were captured with an angular step size of 0.2° over 180° of rotation. The exposure time was 1 s. The distance between the detector and the sample was adjusted to 3.5 cm. Twenty light-field images and five dark-field images were also collected during each acquisition procedure to correct the electronic noise and variations in the X-ray source brightness.

### Comparison of the projection image

To compare the absorption contrast between the stained neuron and surrounding background from the GCM group and M-GCM group, we selected three corresponding slices from each animal for comparison (*n* = 3 × 8). A stained neuron at a similar location (near the central canal) from the corresponding slices was selected for comparison. The projection images were processed and analyzed using the Image-Pro Plus (IPP) software program (version 6.0; Media Cybernetics. Bethesda, MD, USA). The gray value (intensity) data were exported from the IPP software.

### 3D image reconstruction and quantitative determination

All projected tomographic images were transformed into 8-bit slices using the software (Phase-sensitive X-ray Image processing and Tomography Reconstruction, PITRE) developed by the SSRF to perform a direct filtered back-projection algorithm (Chen et al. 2012). Then, all the slices were processed and quantified by Amira software (version 6.01, FEI, USA) (Ian et al. 2016). Depending on the magnitude of X-ray absorption by the neuron, differences in the gray values among tissues were determined. To quantify the neuronal network, we randomly selected the space of 450 × 250 × 1000 μm at the thoracic spinal cord for neuronal network analysis. To compare the neurite length of neurons between the GCM group and M-GCM group, two representative neurons located at the ventral horn (motor neuron) were selected from each mice (*n* = 2 × 8). And all selected neurons had similar soma volume. Neurite length was identified using Image-Pro Analyzer 3D (version 7.0, Media Cybernetics, Inc., Bethesda, MD, USA) (Yang et al. 2013).

### Statistical analysis

All quantitative data are presented as the mean ± standard deviation. All analyses were carried out using SPSS version 24.0 (IBM Corp., Armonk, NY, USA), Groups of data (background gray values, artefact number, gray level deviation of neuronal sites and background, and length of a single neuron neurites) were compared using Student’s *t* test, and *P* values less than 0.05 were considered to indicate statistical significance.

## Results

### M-GCM achieved less background staining, fewer artefacts, and less stained incomplete vasculature compared to the GCM

**Fig. 2:**
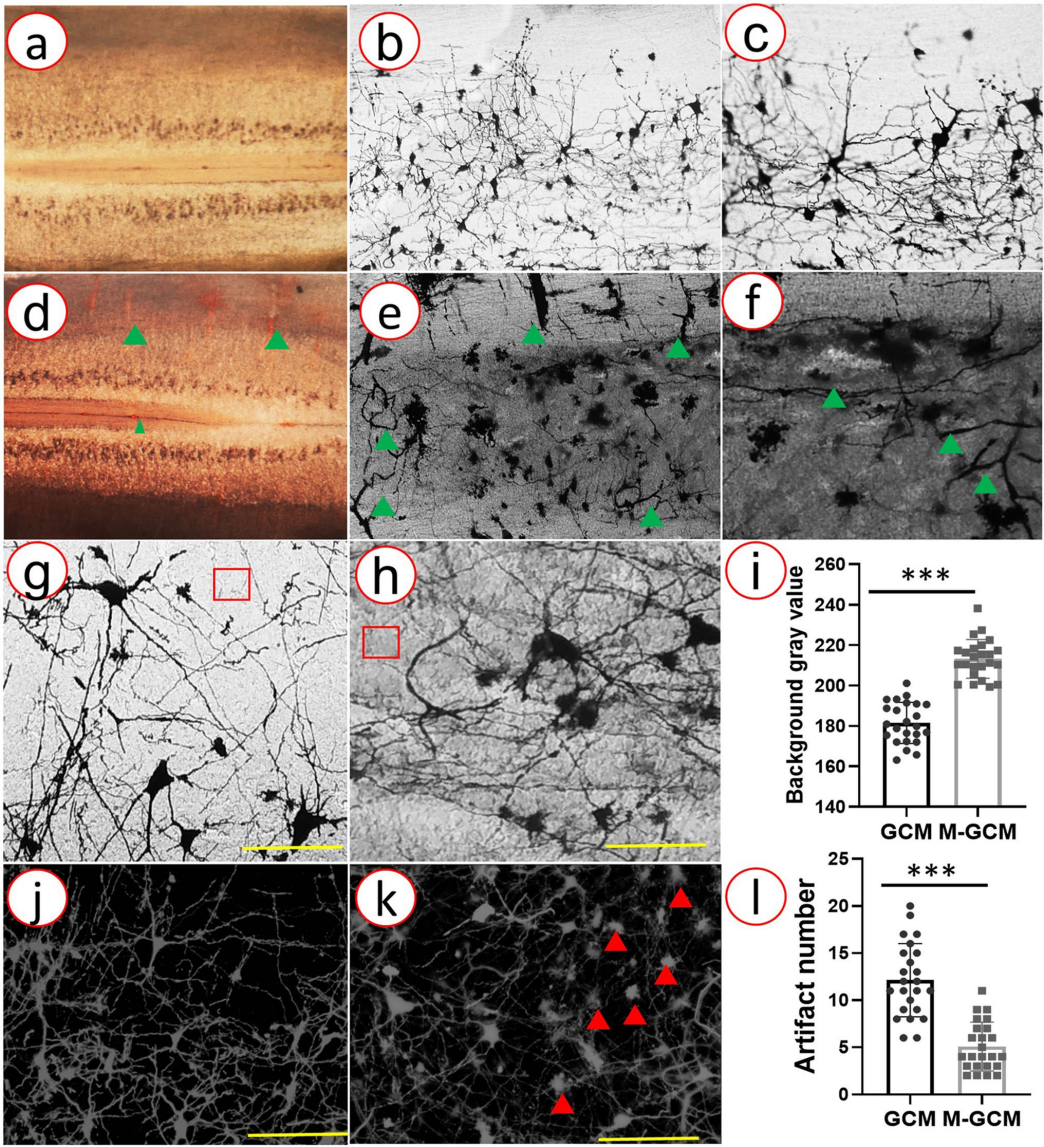
Comparison of photomicrographs from the GCM group and M-GCM group. **a**–**c** Representative images of the fresh and stained spinal cord tissue M-GCM group (**a**, **b**, and **c** are 4×, 10×, and 20× image, respectively). **d**–**f** Representative images of the fresh and stained spinal cord tissue GCM group (**d**, **e**, and **f** are 4×, 10×, and 20× image, respectively). Red arrows indicate the artefacts and the green arrows indicate the incomplete vascular structure. **g** Representative 60 × image shows low background staining in the M-GCM group. **h** Representative 60 × image shows high background staining in the GCM group. **i** A histogram illustrating M-GCM has significantly less the gray value of background compared to that of GCM. **j** Reconstructive 3D images of neuronal networks in the M-GCM group. **k** Reconstructive 3D images of neuronal networks in the GCM group. **l** A histogram illustrating M-GCM has significantly artefact in tissue compared to that of GCM. ***P < 0.0001, Student’s *t* test was used to determine the statistical significance of the differences [scale bar: 120 μm (**g**, **h**) and 250 μm (**j**, **k**)]

In the GCM group, the residual blood, and Solution A/B left within the spinal cord tissue impacted Golgi staining, leading to high background staining, artefacts, and incomplete vasculature. In the M-GCM group, we found that blood was removed by ACSF perfusion, which led to a few stained incomplete vasculatures, as showed in Fig. 2a–c. In contrast, much blood remained in spinal cord vessels, which results in much stained vasculature in the GCM group (Fig. 2d–f). Besides, the photomicrographs from the M-GCM group and GCM present different degrees of background staining (Fig. 2g, h). The background gray value (213.26 ± 19.58) of the M-GCM group was significantly higher than that (181.52 ± 10.17) of the GCM group (*P* < 0.0001) (Fig. 2i). Moreover, the 3D images of the spinal cord gray matter from two groups are shown in Fig. 2j, k. The mean number of artefacts in the 3D region from the GCM group and the M-GCM group was 12.12 ± 3.89 and 5.08 ± 2.59, respectively. Artefacts in the M-GCM group were observed to be significantly less (red arrows indicate in Fig. 2k) than the M-GCM group (*P* < 0.0001) (Fig. 2l). According to the above, slow perfusion with ACSF largely removed blood in the vessels and diminished the interference of vasculature. To sum up, the M-GCM group achieved a more clear background, fewer artefacts, and less incomplete vasculature compared to the GCM group.

### M-GCM increased the refractive index of the stained spinal cord tissue and achieved high-resolution 3D imaging of neuronal network

**Fig. 3:**
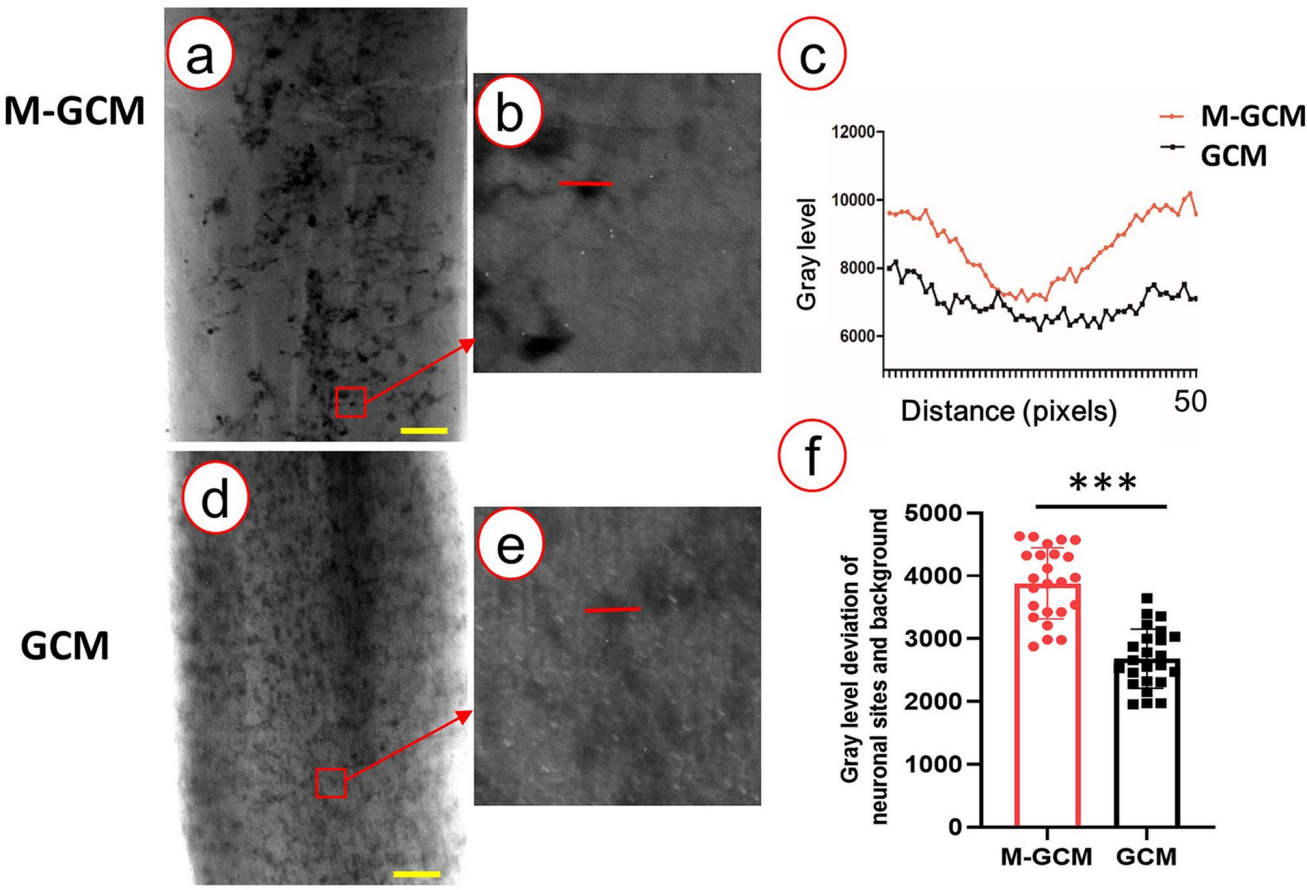
Projection images detected by a high-resolution SRμCT. **a** A representative projection image of a stained spinal cord from the M-GCM group. **b** Local magnification of the region of interest is denoted by the red frame in **a**. **d** A representative projection images of a spinal cord from the GCM group. **e** Local magnification of the region of interest is denoted by the red frame in **d**. **c** Profile of the grey value along the red line in **b** and **e**. **f** A histogram illustrating M-GCM achieved a significantly higher contrast between the neuronal site and surrounding background compared to GCM did. ***P < 0.0001, Student’s *t* test was used to determine the statistical significance of the differences (scale bar: 200 μm)

**Fig. 4:**
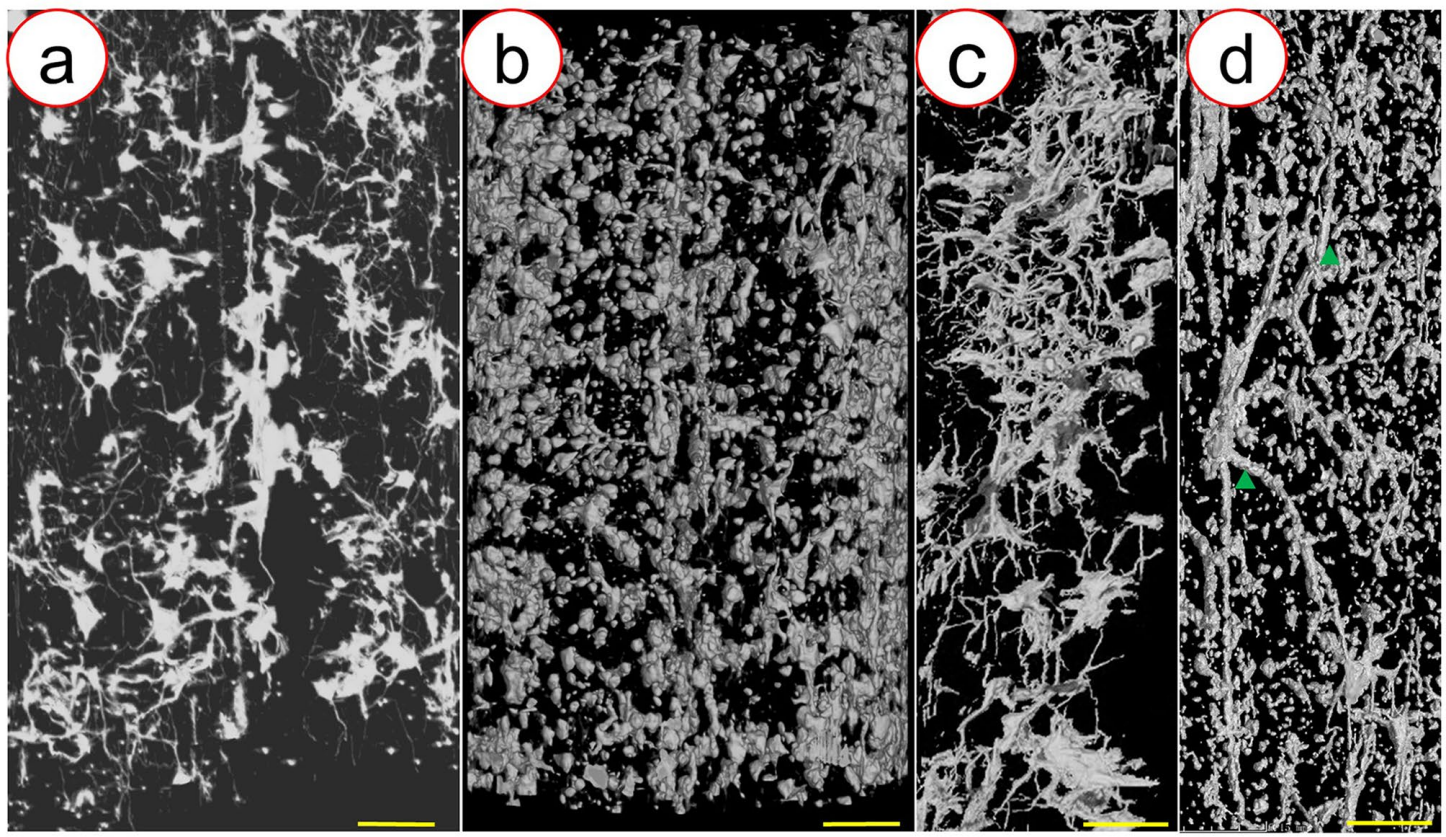
3D images of the neuronal network by SRμCT. **a**, **c** A representative 3D image of the neuronal network from the M-GCM group. **b**, **d** A corresponding 3D image of the neuronal network from the GCM group. The green arrows indicate the incomplete vascular structure [scale bar: 250 μm (**a**, **b**) and 100 μm (**c**, **d**)]

In an attempt to achieve 3D imaging of the neuronal network of the spinal cord, the stained spinal cord tissue from the two groups was used for SRμCT scanning. The representative projection images from the M-GCM group and GCM group are shown in Fig. 3a, d, respectively. According to the results, the M-GCM group increased the refractive index of spinal cord tissue compared to the GCM group. The gray level of the line profiles (red line profile) marked in (Fig. 3b, e) was illustrated in (Fig. 3c). In the M-GCM group, the gray level of the unstained area (background) was significantly higher than that of the stained area (the location of the neuron). In contrast, the gray level of the unstained area was similar to that area of the neuronal site in the GCM group. The deviation of the neuronal sites and background in the M-GCM group was 3800.96 ± 565.60, which was significantly more than that (2860.50 ± 472.12) in the GCM group (*P* < 0.001) (Fig. 3f). Therefore, it was difficult to distinguish the whole neuron and background in the GCM group but M-GCM achieved better. In addition, after these stained spinal cord tissues were detected by SRμCT, numerous 3D neurons could be clearly imaged in the M-GCM group (Fig. 4a). In contrast, there was a large number of artefacts that were difficult to delineate (Fig. 4b) in the GCM group. It was also challenging to outline the entire 3D neuron in the GCM group. 3D images from the GCM group (Fig. 4d) were plagued with more incomplete vasculature compared with 3D images from the M-GCM group (Fig. 4c). According to the above comparison, the M-GCM was further helpful for achieving sufficient contrast to resolve neurons within the tissue and visualizing the 3D neuronal architecture.

### M-GCM presented more detailed features of the neuron than the GCM in 3D imaging

**Fig. 5:**
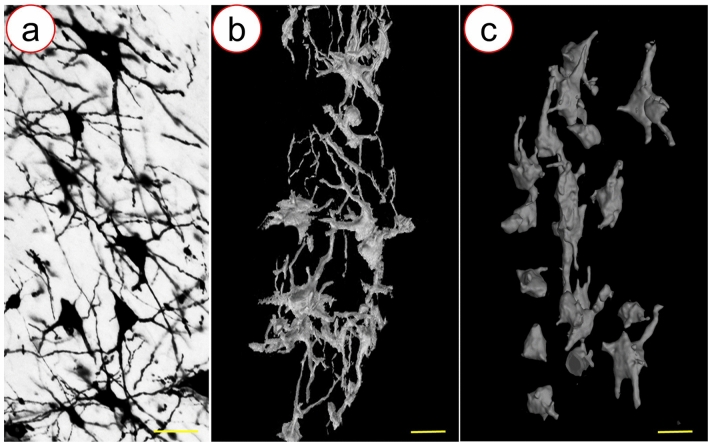
Images of the neuronal network in the ventral horn of the mouse spinal cord. **a** Photomicrograph of the neuronal network in the ventral horn. **b** Digital 3D image of the neuronal network in the ventral horn from the M-GCM group. **c** Digital 3D image of the neuronal network in the ventral horn from the GCM group (scale bar: 50 μm)

**Fig. 6:**
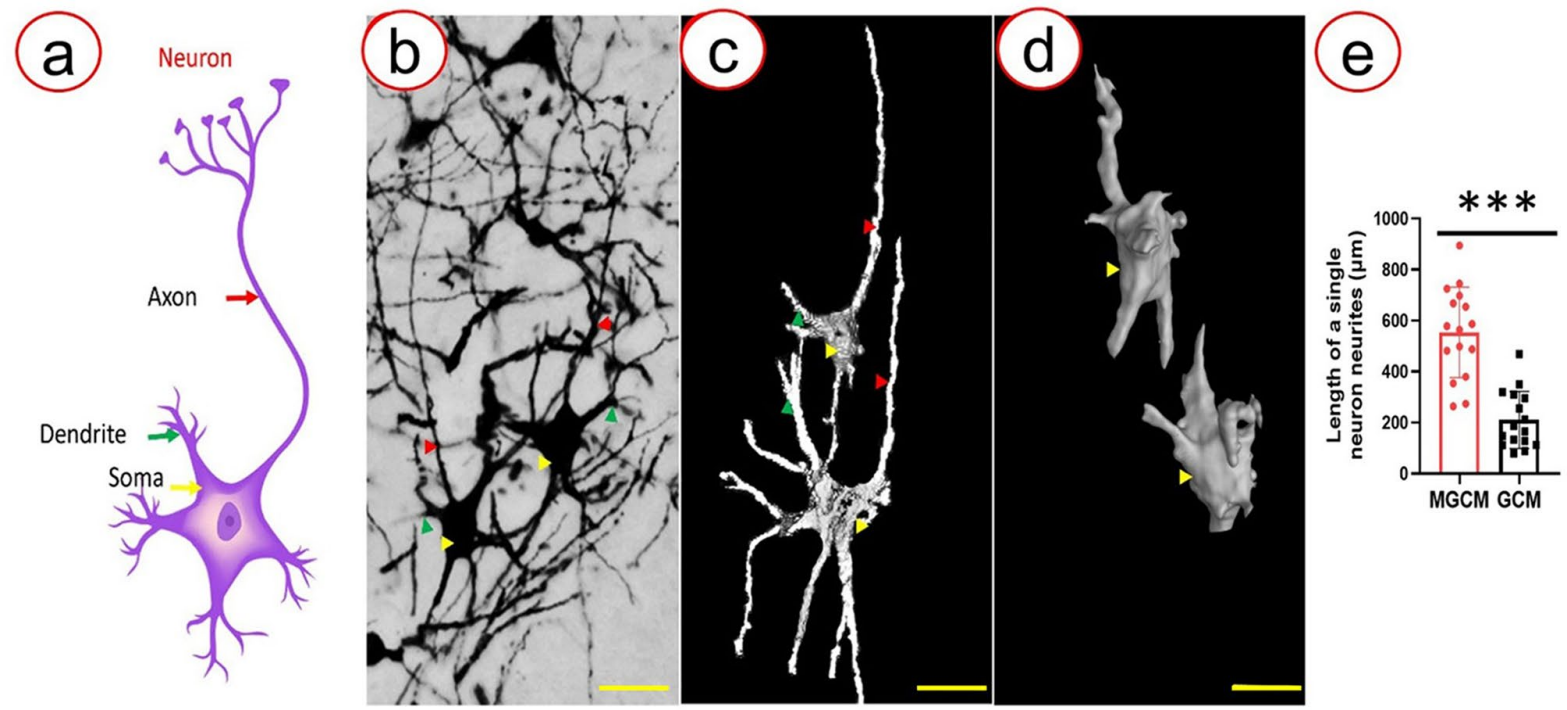
Images of motor neurons in the mouse spinal cord. **a** Schematic depiction of a neuron. **b** Photomicrograph of the motor neurons. **c** 3D reconstructive motor neurons from the M-GCM group. **d** 3D reconstructive motor neurons from the GCM group. Red arrows indicate the axons, green arrows indicate dendrites, and yellow arrows indicate soma. A Histogram illustrating M-GCM visualized longed neurite than GCM did. ***P < 0.0001, Student’s *t* test was used to determine the statistical significance of the differences (scale bar: 50 μm)

The neurons at the ventral horn were selected to show the detailed neuronal network for both groups. As shown in Fig. 5a, the M-GCM group enabled clear visualization of the neuronal network. All entire 3D neurons with axons, dendrites, and soma constituting the neuronal network could be imaged by SRμCT in the M-GCM group (Fig. 5b). But in the GCM group, the incomplete neurons were present in isolation (Fig. 5c). Furthermore, the M-GCM group is better than the GCM group in showing the detailed features of neurons. Based on our schematic illustration of a neuron and photomicrograph demonstrated in Fig. 6a, b, we found that the entire 3D neuronal architecture was visualized in the M-GCM group, as shown in Fig. 6c. However, in the GCM group, only the soma and a small part of neurites were visualized, and most structure of the dendrite and axon was lacking (Fig. 6d). Also, neurons from the M-GCM group had longer neurite length, which ranged from 273.41 μm to 894.36 μm (553.38 ± 177.71 μm), but neurons from the GCM group had short neurite length, which ranged from 78.97 μm to 469.24 μm (210.01 ± 112.06 μm). The average neurite length of a single neuron in the M-GCM group was significantly longer than that in the GCM group (*P* < 0.001) (Fig. 6e). According to these results, the M-GCM presented more detailed features of the neuron than the GCM did.

### Quantification of the 3D neuronal network in M-GCM

**Fig. 7:**
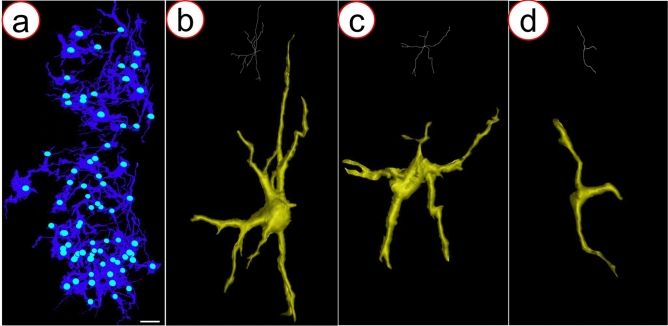
**a** 3D neuronal network at the thoracic mouse spinal cord, quantification for soma number. **b**–**d** Neurons with different shapes, measurements of neurites length. As the figure is shown, the combination of SRμCT and M-GCM has an advantage in the quantification of detailed neuronal architecture (scale bar: 50 μm)

After 3D reconstruction, the 3D neuronal network was displayed in Fig. 7a. The highest spatial resolution that we achieved was 2.0 μm. As Fig. 7a shows, somas could be identified and the number of the somas was 76. Regarding the neurons shown in Fig. 7b–d, the length of neurites was 725.32 μm, 489.71 μm, and 267.27 μm, respectively. The result shows that the combination of SRμCT and M-GCM has an advantage in the quantification of detailed neuronal architecture.

### Analysis of X-ray damage on cellular morphology

**Fig. 8:**
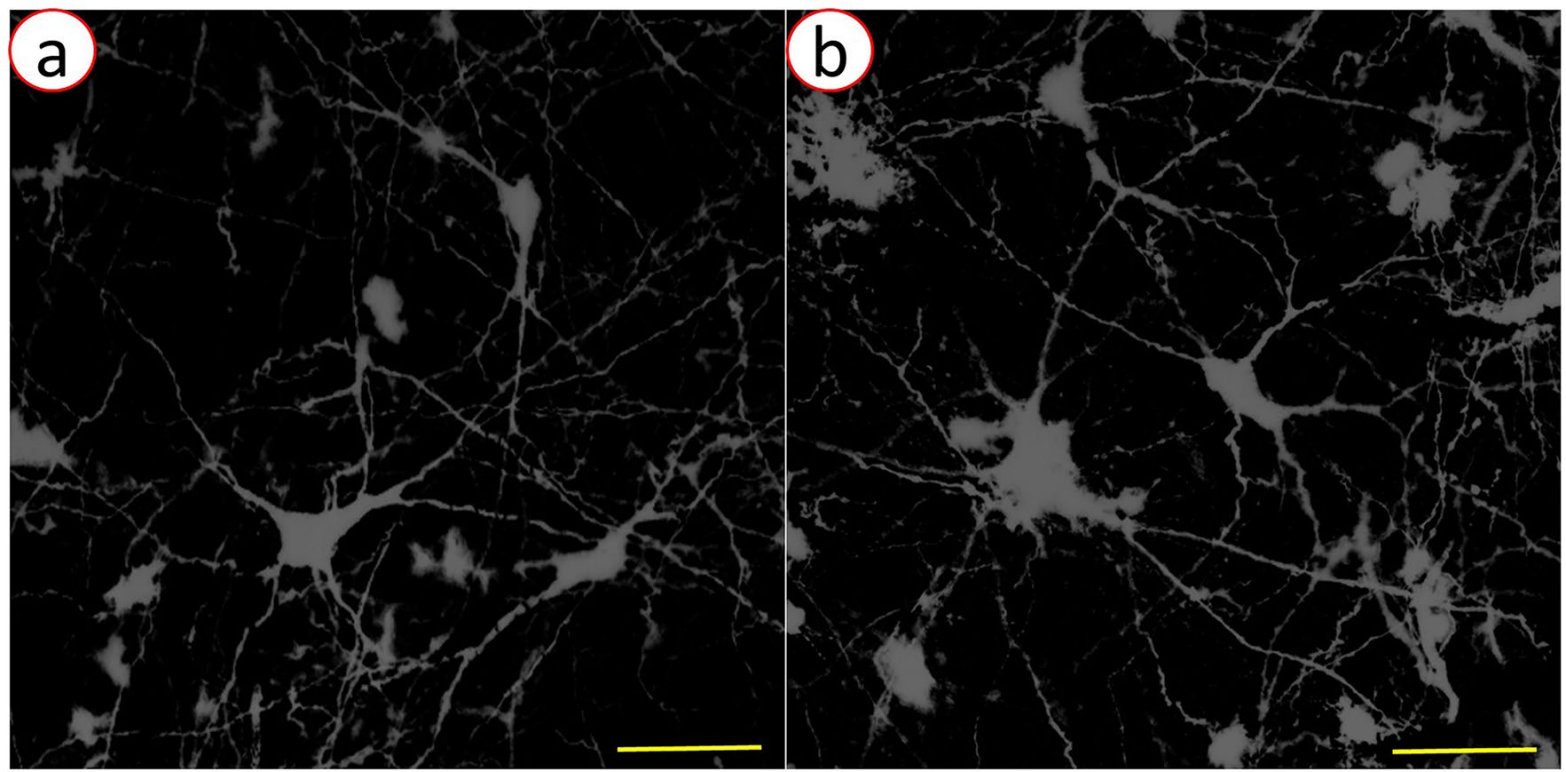
**a** The representative image of stained neurons in the unscanned spinal cord tissue; **b** the representative image of stained neurons in the scanned spinal cord tissue (scale bar: 100 μm)

The representative images of neuronal morphology from unscanned spinal cord tissue and scanned spinal cord tissue were displayed in Fig. 8a, b, respectively. There was no significant difference in neuronal morphology between unscanned tissue and scanned tissue. No obvious X-ray radiation damage on cellular morphology was observed.

## Discussion

In the present study, M-GCM achieved clearer imaging of neuronal morphology in the spinal cord than GCM did. Furthermore, we achieved high-resolution 3D visualization of entire neurons and quantification of the 3D neuronal network in the mouse spinal cord through a combination of SRμCT and the M-GCM. SRμCT show to be a powerful tool that can visualize and evaluate the 3D morphology of Golgi-Cox stained neurons. In contrast to optical 3D techniques, the approach shown here does not require tissue slicing and allows the investigation of numerous neurons within a broad 3D region of the spinal cord. Such a combinatorial method will better serve basic research in neuroscience.

For high-resolution 3D imaging of the neuronal architecture, sample preparation is very important, which will largely influence the image quality. Specific neuron labeling and high contrast between the neuronal site and surrounding background helps to achieve high-resolution 3D imaging of neuronal structure. By far, Golgi staining is still important in neuroscience research due to its whole neuronal labeling characteristic. After years of improvement, the consumption time of Golgi staining has been significantly reduced, and the imaging quality has been increased (Ranjan and Mallick 2010, 2012; Narayanan et al. 2020; Czechowska et al. 2019). However, we find that the GCM still has several shortcomings, such as high background staining, artefacts, and incomplete vasculature labelling (Mizutani et al. 2008; Bentivoglio et al. 2019; Rosoklija et al. 2014). Our study optimized it in spinal cord presentation. First, the current staining frequently results in incomplete vasculature. The residual blood within the tissue will affect the staining, and the neuronal structure will be interfered with the incomplete vascular structure (Mizutani et al. 2008, 2010; Gaballa and Goldman 1999; Monroy-Gomez et al. 2018). However, few studies have mentioned how to improve this issue (Rosoklija et al. 2014). In our study, by infusion of ACSF in the M-GCM, we can effectively diminish the interference of vascular morphology, in contrast to using unperfused fresh specimens for staining in the GCM. Second, background staining and artefact are still an obstacle that reduces the transparency of samples in traditional GCM. During the staining process, residual staining Solution A/B was deposited in the nonneuronal structure areas, which led to inappropriate labeling and opaque tissue background. In current method, we rinsed the tissue in dd-H_2_O multiple times to elute the residual staining solution and achieved more specific neuron labeling on a clearer background. Last, in the GCM, mounting the tissue sections on the slide at the first step was not good for thoroughly clearing the two sides of tissue sections. By contrast, in our M-GCM method, washing the tissue in dd-H_2_O was very helpful in reducing the impact of residual staining solution on imaging quality. Methyl salicylate could help to gain high-grade tissue transparency (Senatorov 2002; Hu et al. 2014). Compare with previous studies that used the GCM for spinal cord tissue staining, we got a better visualization of neurons (Khaw et al. 2020; Hong et al. 2019). Therefore, the M-GCM could better serve morphological research of the neuronal network in the future.

In previous studies, 3D imaging of neurons has been achieved through the combination of Golgi staining and advanced microscopes such as two-photon or confocal microscopy (Kassem et al. 2018; Mancuso et al. 2013). However, the field of vision and the tissue penetration of the microscopy was limited, which severely restricts its use for a large sample. SRμCT is a promising and powerful 3D imaging tool that can help us achieve 3D imaging of larger specimens at the micron level. To achieve 3D imaging of neurons in the mass of the spinal cord, the combination of SRμCT and M-GCM was used in the present study. After many tries, high-resolution 3D imaging of neurons with detailed morphological features could be achieved when the distance between the detector and the sample was 3.5 cm and transmissivity of light was 30–70%. Our results showed that M-GCM significantly increases the refractive index of the spinal cord tissue and reduced the artefacts and the incomplete vascular structure on the 3D images. Compared with previous studies, the current method achieved more detailed 3D imaging of neuronal architecture in the mouse spinal cord (Fratini et al. 2015; Bukreeva et al. 2017; Cedola et al. 2017). Furthermore, we achieved a quantitative assessment of the neuronal network, including soma density and axon length. No X-ray radiation damage to cellular morphology was observed in our study. In recent years, a study reported that they achieved 3D imaging of the neuronal architecture of the mouse brain through a combination of SRμCT and Golgi staining (Fonseca et al. 2018). However, their sample preparation procedure of brain tissue was different from the present study. In addition, this combinatorial method has not been used in 3D imaging of neuronal networks in the spinal cord. With our method applied in the research of neuronal morphology, it would allow us to assess the neuronal network in large structures, but do not require tissue sectioning. Accordingly, the new technique presented here will play an essential role in understanding normal and pathological neural networks and quantifying their characteristics.

## Conclusion

In conclusion, we modified Golgi-Cox impregnations for better visualization of the neurons and proved that the combination of SRμCT and M-GCM is a powerful method for 3D imaging of detailed neuronal architecture in the mass of the spinal cord.

## Supplementary Information

Below is the link to the electronic supplementary material.

Flow chart of the M-GCM and GCM group (PDF 153 KB)

## Data Availability

The datasets generated and/or analyzed during the current study are available from the corresponding author on reasonable request.

## Abbreviations

2D: Two-dimensional
3D: Three-dimensional
SRμCT: Synchrotron radiation micro-computed tomography
M-GCM: Optimized Golgi Cox method
GCM: Golgi-Cox method
dd-H2O: Double-distilled water
ACSF: Artificial cerebrospinal fluid
