# Supplementary figures and images for: A combinatorial method to visualize the neuronal network in the mouse spinal cord: combination of a modified Golgi-Cox method and synchrotron radiation micro-computed tomography
Source: Histochem Cell Biol. 2021 Jan 4;155(4):477–89. doi: 10.1007/s00418-020-01949-8 (PMC8062354; doi:10.1007/s00418-020-01949-8)

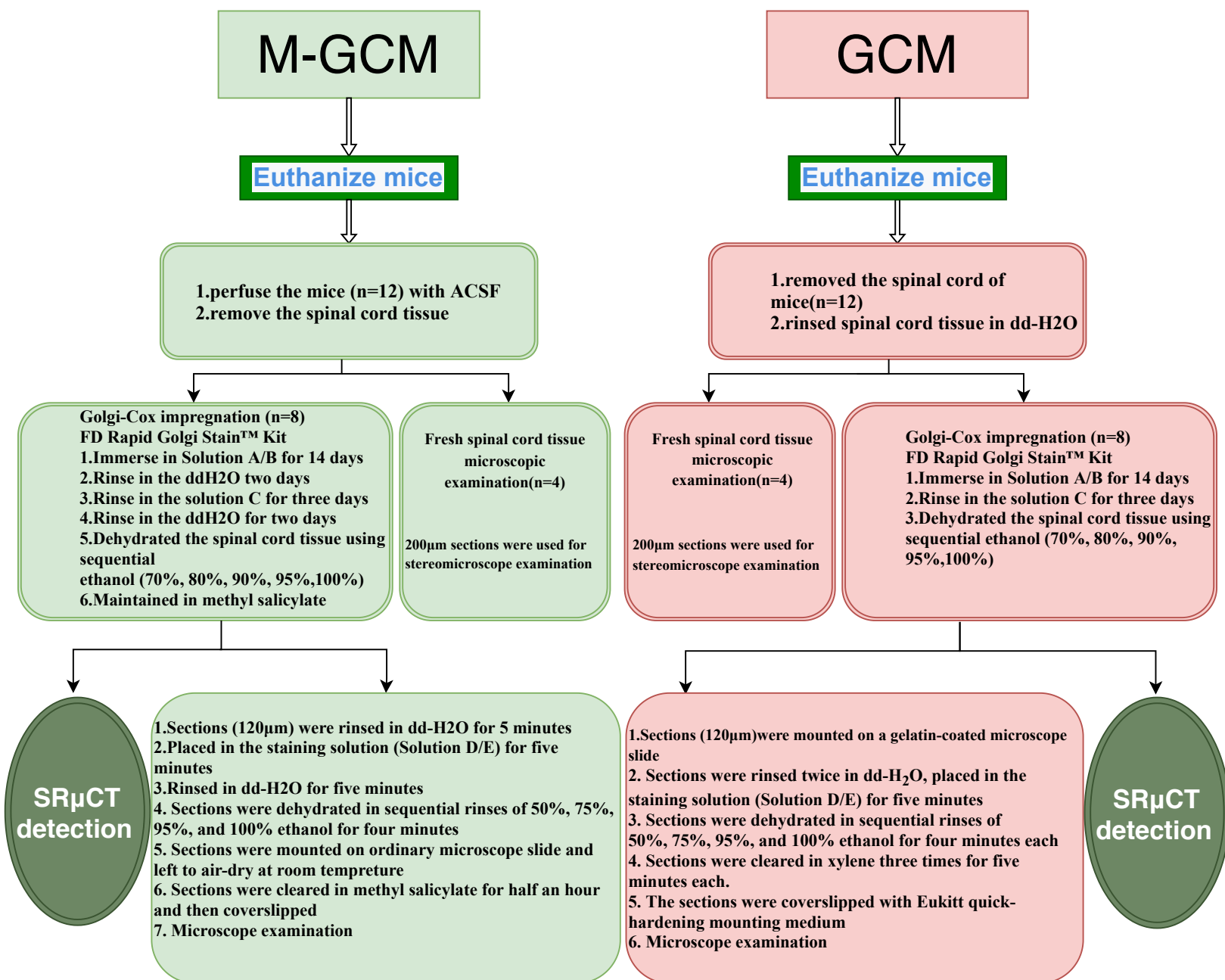

Supplement: Supplementary file 1 — Flow chart of the M-GCM and GCM group (PDF 153 KB) [file 418_2020_1949_MOESM1_ESM.pdf]
